# Sentinel node biopsy using indocyanine green in oral/oropharyngeal cancer

**DOI:** 10.1186/s12957-015-0691-6

**Published:** 2015-09-17

**Authors:** Hanwei Peng, Steven J. Wang, Xiaohua Niu, Xihong Yang, Chongwei Chi, Guojun Zhang

**Affiliations:** Department of Head and Neck Surgery, Cancer Hospital of Shantou University Medical College, No.7 Raoping Road, Shantou City, Guangdong Province 515031 China; Department of Otolaryngology—Head and Neck Surgery, University of California, San Francisco, San Francisco, CA USA; Intelligent Medical Research Center, Institute of Automation, Chinese Academy of Sciences, Beijing, China; Breast Cancer Center, Cancer Hospital of Shantou University Medical College, Shantou, Guangdong China; Current address: Key Lab of Major Obstetrics Disease of Guangdong Province, The Third Affiliated Hospital of Guangzhou Medical University, Guangzhou, Guangdong China

**Keywords:** Sentinel node biopsy, Indocyanine green, Near-infrared fluorescence, Oral neoplasms, Oropharyngeal neoplasms, Methylene blue

## Abstract

**Backgrounds:**

Radioactive tracer-based detection has been proposed as a standard procedure in identifying sentinel nodes for cN0 oral/oropharyngeal carcinoma. However, access to radioactive isotopes may be limited in some surgical centers, and there is potential risk of the radioactive tracers to the operators. This study was designed to evaluate the feasibility of near-infrared fluorescence imaging with indocyanine green combined with blue dye mapping in sentinel node biopsy for cN0 oral/oropharyngeal carcinoma.

**Methods:**

Twenty-six cases of previously untreated oral/oropharyngeal carcinoma staged cT1-2N0M0 were enrolled in this study. One milliliter of indocyanine green (5 mg/ml) and 1.5 ml of methylene blue (1 mg/ml) were injected sequentially around the primary tumor in a four-quadrant pattern before skin incision. After elevation of the platysma flap and posterior retraction of the sternocleidomastoid muscle, fluorescence images were taken with a near-infrared detector, with special attention paid to any blue-dyed lymph nodes. Lymph nodes identified first with fluorescent hot spots with or without blue dye were defined as sentinel nodes, and they were harvested and sent for pathologic study.

**Results:**

Sentinel nodes were successfully harvested in all 26 cases. The number of sentinel nodes (SNs) per case varied from 1 to 9, with an average of 3.4. Routine pathology demonstrated occult metastasis exclusively in SNs in four cases (15.4 %). No tracer-associated side effects occurred in this series.

**Conclusions:**

Near-infrared imaging using indocyanine green combined with methylene blue mapping is a feasible and reliable new method for SN biopsy in cN0 oral/oropharyngeal carcinoma.

## Background

Management of the clinically negative neck (cN0) in oral/oropharyngeal squamous cell carcinoma remains a dilemma. Because there is no diagnostic test available that can accurately evaluate cervical lymph node metastatic status, management options for this situation have included elective neck dissection or watchful waiting [1–3]. However, studies demonstrate that 20–40 % of cN0 patients have occult cervical metastases and will inevitably develop cervical recurrence if a watchful waiting policy is followed; on the other hand, the 60–80 % of patients who have truly negative neck nodes will risk unnecessary injury if an elective neck dissection is performed. Heretofore, selective neck dissection (SND), a procedure that is less invasive than radical neck dissection or modified neck dissection, is widely accepted as the gold standard for management of cN0 neck of oral/oropharyngeal cancer [1, 4–8].

In the past two decades, sentinel node biopsy (SNB) has been proposed as a promising minimally invasive procedure for management of the cN0 neck of oral/oropharyngeal carcinoma [1, 9, 10]. SNB allows the surgeon to identify and harvest the upper echelon lymph nodes that drain the site of a primary malignancy. Furthermore, SNB allows the pathologist to focus on a few lymph nodes and make further pathologic analysis of both clinically occult micro- and macro-metastases. Use of this technique may avoid overtreatment of oral/oropharyngeal carcinoma with pathologically N0 neck.

SNB is a more minimally invasive procedure than SND, and it has higher sensitivity and specificity for evaluating the metastatic status of the cN0 neck in oral/oropharyngeal carcinoma compared to traditional physical examination and imaging scans (CT, MRI, or PET/CT) or image-guided FNA [1, 9, 10]. Although SNB has been administered as a routine procedure in breast cancer, skin melanoma, and vulvar carcinoma in many centers worldwide, its use in head and neck mucosal cancers is much less common, likely because of challenges with regard to sentinel node (SN) detection techniques when applied to the head and neck [11–14]. Preoperative lymphoscintigraphy and intraoperative *γ* probe detection or blue dye mapping is the most commonly used technique for SNB in head and neck mucosal cancers [7, 12]. However, the access of radioactive isotopes is limited in many surgical units, and there is potential risk of the radioactive tracers to the operators. We have previously performed studies of SNB in oral tongue cancers using preoperative lymphoscintigraphy combined with intraoperative Methylene blue dye mapping or with intraoperative γ probe and achieved encouraging results [15–18]. However, because of the inconvenience of the procedure and potential hazards of the radioactive tracer, we attempted to look for an alternative technique which would be accurate, convenient, and safe.

Near-infrared fluorescence (NIF) imaging is a newly developed SNB technique firstly introduced by Kitai et al. in 2005 [19]. Subsequent reports of its successful use in gastric, gynecologic, lung, esophagus, as well as skin cancer are found in the literature [20–22]. To our knowledge, there are only a few pilot studies of SNB with NIF mapping for head and neck mucosal cancer that have been published, which have demonstrated the proof of principle and safety of this technique; however, the largest subject sample size was only 14 cases [23–26]. Clearly, further studies with more patients are needed to establish the feasibility and reliability of NIF imaging with indocyanine green (ICG) for evaluating the cervical lymph node status of cN0 head and neck mucosal cancers. In the current study, we report our results of SNB for 26 cases of cN0 oral/oropharyngeal carcinoma using NIF imaging with ICG combined with methylene blue mapping. Considering the higher possibility of occult metastasis in cT3–4 cases, similar to other studies, we included only cT1-2N0M0 patients in the current study.

## Methods

Patients with previously untreated cT1-2N0M0 oral or oropharyngeal cancer who were to undergo selective neck dissection (levels I–III) or modified radical neck dissection plus radical resection of the primary tumor with or without reconstruction were enrolled in this study. Exclusion criteria were age <18, having a history of previous neck surgery or radiotherapy; pregnancy; lactation; or an allergy to iodine, shellfish, or ICG. Clinical stage was based on physical examination and imaging studies (CT or MRI with contrast).

This study was conducted with the approval of Medical Ethics Committee of Cancer Hospital of Shantou University Medical College and was performed in accordance with the ethical standards of the Helsinki Declaration of 1975. Written informed consent was obtained from all patients before SNB.

SN mapping was performed using the Optical Molecular Imaging Operation Navigation System (OMIONS, Zhongke Medical Imaging Technology Co. Ltd., Anhui, China). The system consists of two wavelength-isolated light sources: a “white” light source, generating 400–650 nm light and a “near-infrared” light source, generating 760 nm light. Color video and NIR fluorescence images are simultaneously acquired and displayed in real time using custom optics and software that separate the color video and NIF images. The imaging head is attached to a flexible double-joint arm, which permits positioning of the imaging head virtually anywhere over the surgical field [27].

**Fig. 1:**
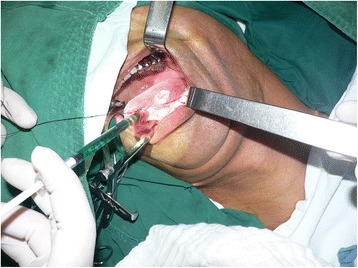
One milliliter of indocyanine green at a concentration of 5 mg/ml was injected around the tumor in a four-quadrant pattern

**Fig. 2:**
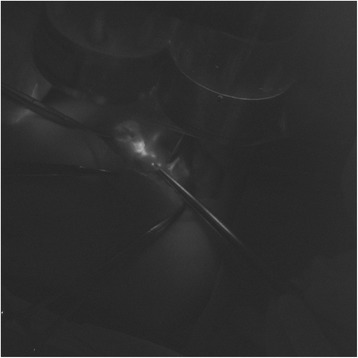
After exposure of the subplatysmal plane in the neck and retraction of the sternocleidomastoid muscle posteriorly, fluorescent hot spots appeared under “near-infrared” light source

**Fig. 3:**
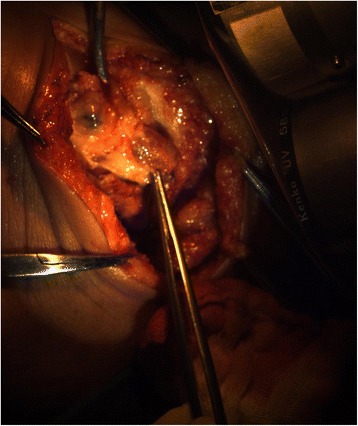
Color image of the sentinel nodes under “white” light source shows visible blue dye

**Fig. 4:**
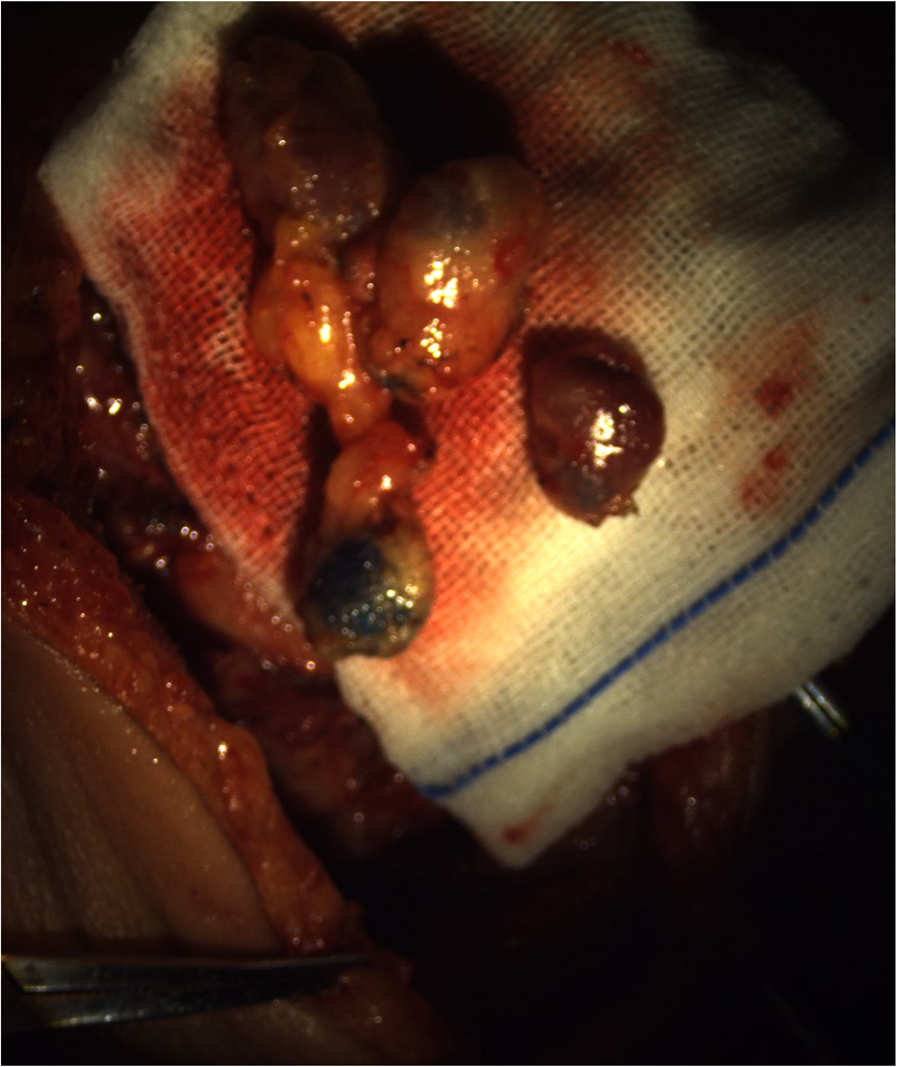
Ex vivo sentinel nodes with or without blue dye

**Fig. 5:**
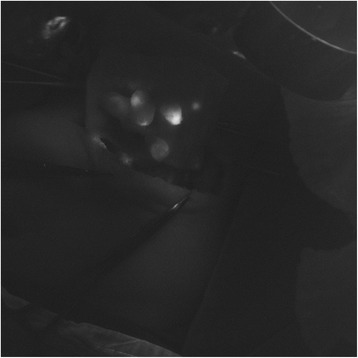
Ex vivo confirmation using OMIONS showed all sentinel nodes had fluorescent hot spots

The ICG (Dandong, China) was dissolved at a concentration of 5 mg/ml, and the methylene blue (Beijing, China) was prepared at a concentration of 10 mg/ml. One milliliter of ICG was injected around the tumor in a four-quadrant pattern (Fig. 1); 1.5 ml of methylene blue was then injected in the same manner. Immediately after injection of the tracers, a standard incision was made and the platysma flap was elevated. After exposure of the subplatysmal plane in the neck and retraction of the sternocleidomastoid muscle posteriorly, the surgical field was measured for fluorescent signal using the OMIONS. During neck dissection, measurement of fluorescence was performed every 5 to 10 min until hot spot(s) appeared on the screen. Special attention was paid to the blue-dyed lymphatics and lymph nodes. The first appearing lymph nodes with NIF hot spots with or without visible blue dye were defined as SNs and were resected for further confirmation under the OMIONS (Figs. 2, 3, 4, and 5). Neck dissection was continued after harvest of the SNs; any blue-dyed lymphatic at this point was considered to be second station lymph nodes rather than SNs, and they were separately sent for pathologic analysis. The resection of the primary tumor was subsequently performed by standard procedure; reconstruction of the defect was done as indicated. The harvested SNs were bisected along the long axis through their hilum; one half was fixed in formalin, embedded in paraffin, and sent for routine pathologic examination; the other half was deposited in liquid nitrogen for further investigation. All non-SNs were examined by routine histopathological analysis.

## Results

### Patient and tumor features

Clinical data of the patients are detailed in Table 1. A total of 26 consecutive patients with squamous cell carcinoma of the oral cavity (*n* = 19) or oropharynx (*n* = 7) and a clinical stage of T1-3N0M0 were enrolled in this study from July 2014 to December 2014. Median patient age was 60.5 years (range 43–77 years). Fifteen patients had cT1 primary, eleven patients had cT2. Location of primary tumor included oral tongue (*n* = 14), buccal mucosa (*n* = 3), palatoglossal arch (*n* = 3), base of the tongue (*n* = 3), hard palate (*n* = 2), and soft palate (*n* = 1). All patients underwent a radical resection of the primary tumor with negative surgical margins. Of the 26 patients, eleven needed a surgical reconstruction (radial forearm flap, *n* = 7; infrahyoid myocutaneous flap, *n* = 2; local mucosal flap, *n* = 1; skin graft, *n* = 1), whereas primary closure was possible for the remaining patients. Ipsilateral SND was performed in 20 patients, modified radical neck dissection (MRND) was performed in 5 patients, and bilateral SND was performed in 1 patient; selection of an appropriate ND type was based on clinical indications. The sentinel node excision was performed prior to the reconstructive surgery; SNB did not have any impact on reconstruction. There were no complications associated with the technique. SNB added an average of about 15 min to the total operation time compared with a standard neck dissection procedure.

**Table 1:** Clinical data of the patients enrolled

Pt No.	Age	Sex	Primary site	cTNM	ND type	Reconstruction	pTNM	SN site	SN no. (p+/total)	Other LN no.
1	63	M	Palate	T1N0M0	SND	Local mucosal flap	T1N0M0	IIa	0/2	0/12
2	55	F	PGA	T1N0M0	MRND	RFAF	T1N2M0	IIa, III	2/3, 0/2	0/19
3	51	F	Tongue	T1N0M0	MRND	None	T1N0M0	IIa	0/6	0/22
4	59	M	BOT	T2N0M0	SND	None	T3N0M0	IIa	0/2	0/25
5	72	M	Buccal	T2N0M0	SND	None	T2N0M0	Ib	0/1	0/9
6	71	F	Tongue	T2N0M0	SND	None	T3N0M0	IIa	0/3	0/24
7	57	M	Buccal	T2N0M0	SND	None	T3N1M0	Ib	1/1	0/17
8	55	M	Tongue	T1N0M0	SND	IHMCF	T1N2MO	Ib, IIa	1/2, 2/3	0/26
9	66	F	Tongue	T1N0M0	SND	None	T1N0M0	IIa	0/3	0/24
10	49	M	PGA	T1N0M0	MRND	RFAF	T1N0M0	IIa	0/9	0/17
11	56	M	Tongue	T1N0M0	SND	None	T1N0M0	Ib, IIa	0/1, 0/2	0/13
12	45	F	Palate	T2N0M0	SND	RFAF	T2N0M0	IIa	0/1	0/14
13	72	M	PGA	T1N0M0	MRND	RFAF	T1N0M0	IIa	0/5	0/19
14	53	F	Tongue	T1N0M0	SND	None	T1N0M0	Ib, IIa	0/2, 0/2	0/12
15	73	M	Buccal	T1N0M0	SND	Skin graft	T1N0M0	Ib	0/6	0/12
16	47	M	BOT	T1N0M0	bi-SND	IHMCF	T1N0M0	Ia	0/1	0/30
17	75	F	Tongue	T1N0M0	SND	None	T1N0M0	IIa	0/2	0/9
18	73	M	Soft palate	T1N0M0	SND	RFAF	T1N0M0	IIa	0/1	0/11
19	77	F	Tongue	T2N0M0	SND	None	T2N0M0	Ia, IIa	0/2, 0/3	0/17
20	55	F	Tongue	T2N0M0	MRND	None	T3N0M0	Ia, III	0/2, 0/2	0/39
21	64	M	Tongue	T1N0M0	SND	None	T1N1M0	Ib,IIa	1/1, 0/2	0/20
22	56	M	BOT	T2N0M0	SND	RFAF	T2N0M0	IIa, III	0/1, 0/3	0/18
23	76	F	Tongue	T2N0M0	SND	None	T2N0M0	IIa	0/1	0/29
24	50	F	Tongue	T2N0M0	SND	None	T2N0M0	Ib, IIa	0/2, 0/2	0/20
25	68	F	Tongue	T2N0M0	SND	None	T2N0M0	Ia, IIa	0/1, 0/2	0/17
26	62	F	Tongue	T1N0M0	SND	None	T1N0M0	Ia, III	0/1, 0/3	0/23

*Abbreviations*: *ND* neck dissection, *SN* sentinel node, *LN* lymph node, *p+* pathologically positive, *M* male, *F* female, *SND* selective neck dissection, *bi-SND* bilateral selective neck dissection, *cSND* contralateral selective neck dissection, *PGA* palatoglossal arch, *MRND* modified radical neck dissection, *RFAF* radial forearm flap, *BOT* base of the tongue, *IHMCF* infrahyoid myocutaneous flap

At least 1 SN was successfully detected in all 26 patients. A total of 88 SNs were harvested, with a SN number per case of 1-9, average of 3.4 SNs. The number of non-SNs was 498 in 26 cases, with an average of 19.2 per case. All the SNs were detected in the ipsilateral neck; anatomic distribution of these 88 SNs was as follows: level IIa 63.6 % (56/88), level Ib 17.0 % (15/88), level III 11.4 % (10/88), and level Ia 8.0 % (7/88).

Routine histological analysis of the neck dissection specimens demonstrated that four patients had occult lymph node metastasis (15.4 %), and all the metastatic tumors were found exclusively in SNs. Nodal classification was elevated from cN0 to pN1 and pN2 both in two patients.

Additional blue-dyed lymph nodes after removal of the SNs were found in 11 patients. All these lymph nodes were free from metastasis under routine pathology.

No side effects associated with ICG injection were observed in this series.

## Discussion

Although multiple studies have demonstrated the potential benefit of SNB in head and neck cancers, only a few centers have used this technique to determine the necessity of neck dissection for cN0 head and neck cancers, due to several possible reasons including the complex lymphatic anatomy of the head and neck and less accessibility of the primary tumor location [28]. With regard to SN detection techniques, preoperative lymphoscintigraphy, intraoperative γ probe detection, with or without blue dye mapping, is now considered the gold standard method [12, 29, 30]. However, several disadvantages of this standard procedure are notable: (1) SNB using radioactive tracers requires a nuclear physician, and both the staff and patient are exposed to radiation although the dose is much lower than the maximum safe limit; (2) the use of radioactive tracer is also associated with the need to safely dispose of any contaminated waste; (3) preoperative lymphoscintigraphy entails an additional procedure for the patient which increases the overall cost of care; furthermore, the lymphoscintigraphy injection is done while patients are awake which may provoke pain and anxiety for some; (4) the so-called shine-through phenomenon handicaps SN detection when the primary site and the possible SNs are in close anatomic proximity, e.g., cancer of the floor of the mouth [5, 12]; and (5) blue dye also has disadvantages, in that blue-dyed SNs can only be identified when the lymphatic or lymph nodes are surgically exposed. Thus, developing a novel alternative to overcome these pitfalls without compromising diagnostic reliability could increase the acceptance and use of SNB for head and neck cancer. We hypothesize that intraoperative NIF imaging with ICG combined with blue dye mapping is a SNB technique that is a potential alternative to the current gold standard of isotope detection.

ICG has been used extensively in the medical field since 1957 [31]. It is now widely used in measurement of liver/renal function, retinal angiography, cardiac output, as well as perforator detection in flap harvest and monitoring [32]. As soon as ICG is infused intravenously, it rapidly binds to plasma proteins and thereby is confined to the vascular space. ICG is removed exclusively by the liver at the rate of 18–24 % per minute with a half-life of 150–180 s [22]. The clinical use of ICG in detection of SN successfully was first introduced by Kitai in breast cancer [19]. To our knowledge, only a few studies on SNB for head and neck mucosal carcinoma using NIF imaging with ICG have been reported in the English literature, and the results show potential feasibility [23–26, 28]. However, further studies are needed to verify the feasibility, efficacy, and optimal protocol for this technique.

Drawing from our previous experience doing SNB for oral cancer using radioactive tracer and blue dye mapping, we performed the current study to evaluate the feasibility and efficacy of SNB using NIF imaging combined with methylene blue dye mapping for oral/oropharyngeal carcinoma. The fluorescent signals were successfully captured after elevation of the platysma flap and posterior retraction of the sternocleidomastoid muscle in all 26 cases. SNs in 15 cases were also visibly blue dyed. In a study using ICG with blue dye in SNB for breast cancer, Hirono et al. defined both fluorescence positive and blue-dyed lymph nodes as SN [33]. However, we surmise that migration of ICG from the primary to the SNs is in the same manner as but faster than that of methylene blue [20], resulting in a proportion of SNs that concentrate only detectable ICG without visually detectable level of methylene blue. All the blue-dyed lymph node(s) identified after fluorescence hot spots were free from metastasis, supporting the rationale of the SN definition used in this series: the lymph node(s) with hot spots detected first were defined as SN. Anatomic distribution of the SNs in the current study was similar to the metastatic pattern of oral/oropharyngeal cancer in our early retrospective study [34]: most frequently in ipsilateral level IIa (63.6 %, 56/88), followed by level I (25.0 %, 22/88), finally level III (11.4 %, 10/88). The average number of SNs harvested in this study was higher than our previous study using radioactive tracer (3.4 per case vs. 2.0 per case) [16, 17].

There are several advantages of this technique compared with standard SNB with radioactive tracers. (1) ICG is a radiation-free tracer and safe, rare side effects have been reported, and special radiation protection is unnecessary. (2) SNB using NIF imaging with ICG is a convenient one-step procedure; patients’ discomfort and anxiety during preoperative injection of the tracer for lymphoscintigraphy can be avoided. (3) The cost of a full dose of ICG is much less than a radiotracer, and the fluorescence imaging device is also less expensive than a γ probe; therefore, as a result of the lower cost, patients in developing countries may also benefit from SNB with ICG. (4) With stimulation of near-infrared light, the fluorescent signal of the ICG was detectable after platysmal flap elevation and posterior retraction of the sternocleidomastoid muscle, making it easy for a surgeon to remove the SNs; as demonstrated in this study, SNs were successfully identified in all 26 cases. (5) SNs harvested with this technique represent the first echelon nodes that drain the primary site. In the current study, we demonstrated that fluorescence positive SNs accurately determined the cervical lymph node metastatic status in all 26 cases, without any false negatives.

In this study, selective or modified radical neck dissections were still performed in all patients. Our results suggest removal of sentinel nodes alone could reliably demonstrate the cervical nodal status for these tumors. If comprehensive neck dissection could be limited to those with positive sentinel nodes, the potential morbidity of this surgical procedure would be avoided and the cost and length of hospital stay might be reduced.

Interestingly, the average number of SNs harvested in our study is higher than other reports [33]. We believe that this may be related to the sensitivity of the OMIONS. Since quantification of the NIF is not possible using the current generation of OMIONS, we harvested all lymph nodes with any visible fluorescent signal.

Another previously reported advantage of SNB with ICG fluorescent imaging in superficial cancers is that it enables visualization of lymphatic drainage from the primary tumor to the regional lymph node basin. Thus, SNs can be identified and resected more rapidly and easily, especially in cases with multiple lymphatic drainage pathways [35]. However, this advantage was not observed in our study. We failed in attempts to measure fluorescent signal transcutaneously in all the first five cases. A possible explanation is that the penetrating thickness through soft tissues of NIF from ICG is 0.5–1 cm [32, 35], whereas oral cavity and oropharyngeal lymphatic channels and SNs are usually embedded in the deep fatty tissue and blocked by either mandible or sternocleidomastoid muscle, making it impossible for the CCD camera to capture the NIF signal transcutaneously.

The inability to detect NIF signal transcutaneously does mean that an initial neck incision of sufficient length must be made to allow exposure of the expected neck levels where the sentinel nodes are likely located, which is an acknowledged drawback of this technique. In comparison, with the radioactive tracer/γ-probe technique, transcutaneous detection allows one to make a small skin incision over the target sentinel nodes. Another drawback of this technique is that shadowless lights in the operating theater give off their own intrinsic NIF beam; therefore, lymphatic navigation must be performed in the dark to minimize the interference with beams produced by the ICG.

## Conclusions

Based on the results of our study, which is the largest series reported to date, we conclude that NIF imaging with ICG to identify the SNs in oral/oropharyngeal cancer is feasible, reliable, and safe. SNB using this technique correctly evaluates the cervical node status as confirmed on routine pathology of complete neck dissection specimens. NIF imaging with ICG is a promising SNB technique for oral/oropharyngeal cancer that deserves further research to refine the technique. Future consideration should be given to a prospective trial comparison of NIF imaging with ICG versus radioisotope techniques for sentinel node identification in oral cancer.

## Abbreviations

BOT: base of the tongue
cN0: clinically negative neck
IHMCF: infrahyoid myocutaneous flap
MRND: modified radical neck dissection
NIF: near-infrared fluorescence
OMIONS: Optical Molecular Imaging Operation Navigation System
PGA: palatoglossal arch
RFAF: radial forearm flap
SNB: sentinel node biopsy
SND: selective neck dissection
